# Multi-level feature fusion network for neuronal morphology classification

**DOI:** 10.3389/fnins.2024.1465642

**Published:** 2024-10-21

**Authors:** Chunli Sun, Feng Zhao

**Affiliations:** MoE Key Laboratory of Brain-inspired Intelligent Perception and Cognition, University of Science and Technology of China, Hefei, China

**Keywords:** cross-attention, feature fusion, multi-level fusion, neuronal morphology, neuron classification

## Abstract

Neuronal morphology can be represented using various feature representations, such as hand-crafted morphometrics and deep features. These features are complementary to each other, contributing to improving performance. However, existing classification methods only utilize a single feature representation or simply concatenate different features without fully considering their complementarity. Therefore, their performance is limited and can be further improved. In this paper, we propose a multi-level feature fusion network that fully utilizes diverse feature representations and their complementarity to effectively describe neuronal morphology and improve performance. Specifically, we devise a Multi-Level Fusion Module (MLFM) and incorporate it into each feature extraction block. It can facilitate the interaction between different features and achieve effective feature fusion at multiple levels. The MLFM comprises a channel attention-based Feature Enhancement Module (FEM) and a cross-attention-based Feature Interaction Module (FIM). The FEM is used to enhance robust morphological feature presentations, while the FIM mines and propagates complementary information across different feature presentations. In this way, our feature fusion network ultimately yields a more distinctive neuronal morphology descriptor that can effectively characterize neurons than any singular morphological representation. Experimental results show that our method effectively depicts neuronal morphology and correctly classifies 10-type neurons on the NeuronMorpho-10 dataset with an accuracy of 95.18%, outperforming other approaches. Moreover, our method performs well on the NeuronMorpho-12 and NeuronMorpho-17 datasets and possesses good generalization.

## 1 Introduction

Neuron classification based on morphological characteristics is essential but challenging due to numerous types, insufficient high-quality reconstructions, and vague definitions of differences among different types (DeFelipe et al., 2013; Armañanzas and Ascoli, 2015; Zeng and Sanes, 2017; Glaser et al., 2019; López-Cabrera et al., 2020). In order to solve these difficulties and realize accurate identification of neurons, researchers are devoted to exploring the efficient representation of neuronal morphology (Laturnus et al., 2020; Sarkar et al., 2013; Wu et al., 2008; Batabyal and Acton, 2018; Basu et al., 2011; Batabyal et al., 2018; López-Cabrera and Lorenzo-Ginori, 2017; Hernández-Pérez et al., 2019).

**Figure 1:**
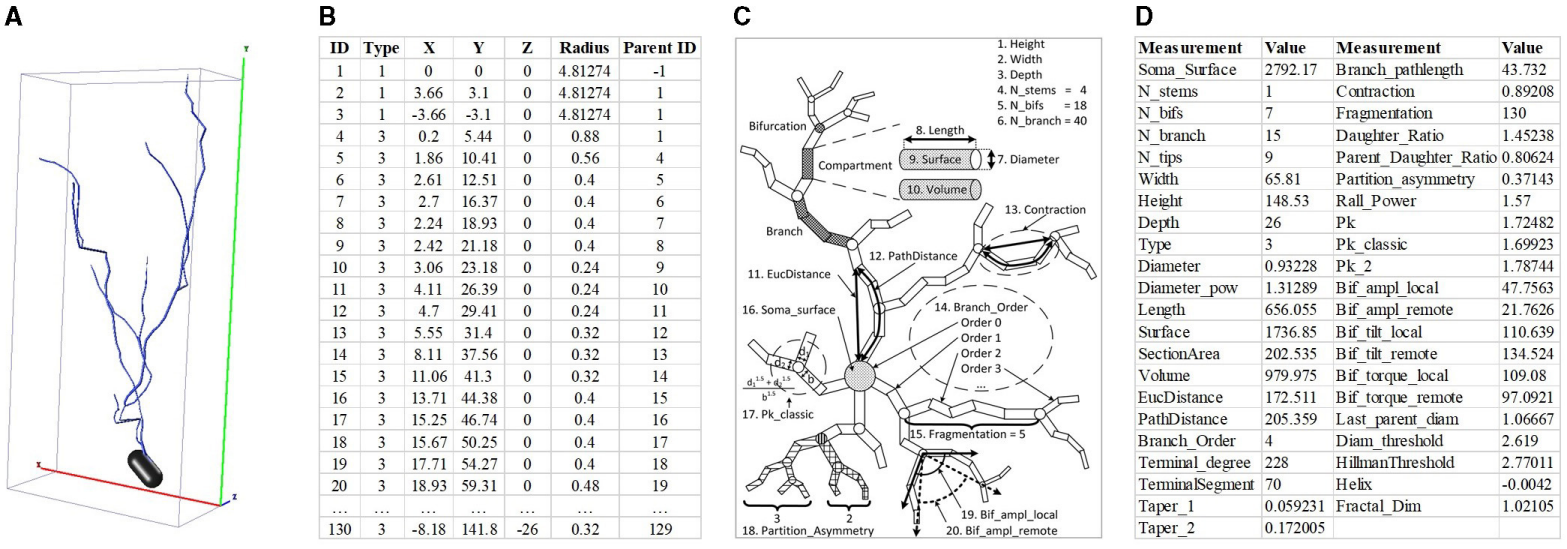
Neuron data in different formats and hand-crafted morphometrics. **(A)** is 3D neuron data and the red, green, and blue represent the X, Y, and Z axis, respectively. **(B)** is the neuron data stored in the SWC format. **(C)** is the definition of hand-crafted morphometrics (Costa et al., 2010) and **(D)** is the 43 hand-crafted morphometrics computed by the L-Measure toolbox (Scorcioni et al., 2008).

Neuronal morphologies typically are represented in two primary formats, namely the 2D or 3D image and SWC format file formats (as shown in Figures 1A, B). The SWC format is low-dimensional and unstructured. It describes neurons as a non-strict binary tree (Zhang et al., 2021). When the neuronal branches under the same bifurcation are exchanged, the neuronal morphology does not vary. Moreover, the length of neuron points is directly related to the sampling rate. This leads to a large variability in the length among different neurons. Conversely, the 2D or 3D image is structured but suffers from high dimensionality (Zhang et al., 2021). These images possess similarities in local features and invariance to translation rotation. Nevertheless, compared to the complexity of neuronal morphology, the number of samples is insufficient. This makes neuronal morphology analysis particularly challenging.

To effectively describe neuronal morphology, the hand-crafted morphometrics (as shown in Figure 1C), such as the number of bifurcations, the number of branches, and the soma surface (Uylings and Van Pelt, 2002; Costa et al., 2010; Wan et al., 2015; Bird and Cuntz, 2019), are designed and widely used. The hand-crafted morphometrics usually measure the neuronal morphology as statistic real numbers, such as mean or standard variance (as shown in Figure 1D). Although hand-crafted morphometrics perform well in characterizing neurons, they would fail to describe neurons with very complex dendrites (Scorcioni et al., 2008). Later, researchers increasingly focus on analyzing advanced and intricate morphological descriptors to fully characterize neurons. Based on the topological and geometrical theories and the tree-like structure of neurons, various morphological descriptors are developed, such as shape descriptor (Sarkar et al., 2013), Sholl analysis (López-Cabrera et al., 2020; Khalil et al., 2022), and Topological Morphology Descriptors (TMD; Kanari et al., 2018).

Recently, motivated by the development of deep learning techniques, many studies utilize neural networks to extract deep features from 3D neuron data (Lin and Zheng, 2018; Zhao et al., 2022; Kanari et al., 2024) or 2D images (Li et al., 2018, 2021; Sun et al., 2023). The sparsity of 3D neuron data makes training 3D neural networks challenging. For example, training 3D networks requires significant computation resources and train time. Furthermore, the variability in the number of neuron points adds complexity to the design of a unified framework for processing 3D neuron data. Since the 2D image describes the full neuronal morphology, the deep features extracted by Convolutional Neural Network (CNN) perform well in depicting the holistic structure of neurons. Moreover, the hand-crafted morphometrics are a set of statistical values, such as mean or standard variance. They fail to describe neurons with complex dendrites, but relatively effectively characterize the local structure of neurons. It is worth noting that combining deep features with hand-crafted morphometrics can significantly boost classification performance, as proved in (Li et al., 2018; Zhang et al., 2021). Therefore, exploring effective strategies for integrating diverse feature representations to enhance neuronal classification performance is of significant importance.

**Figure 2:**
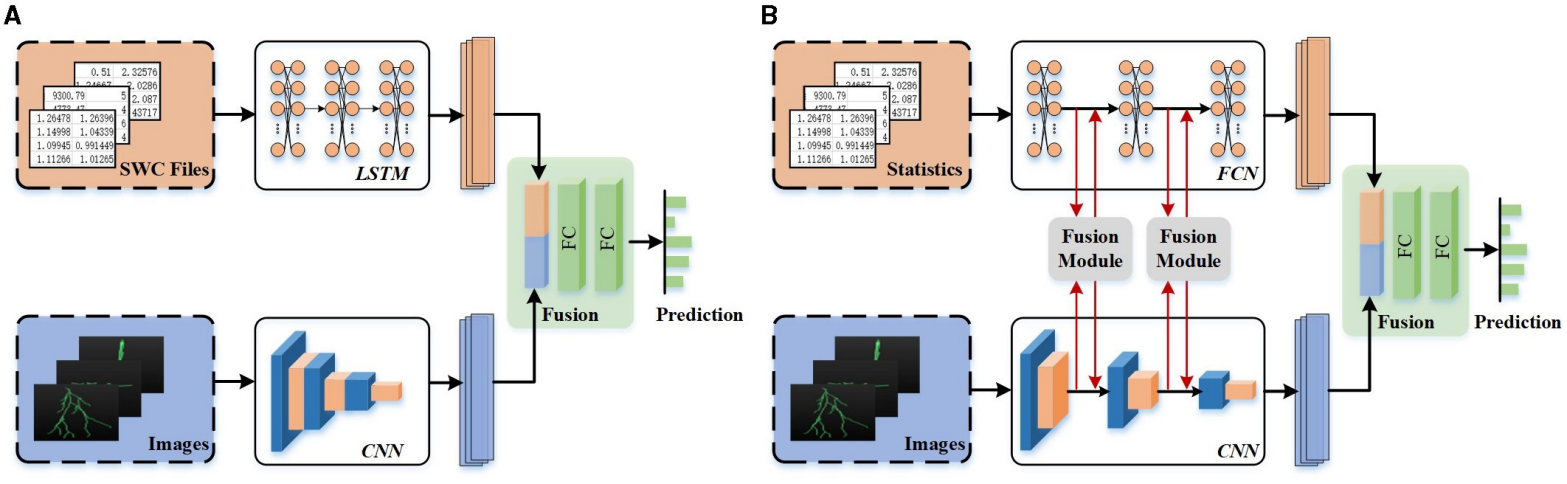
Different fusion networks for neuronal morphology classification. Different features are directly concatenated without any communication (Zhang et al., 2021) **(A)** or fused after information interaction at multi-levels **(B)**.

Many studies recently attempt to integrate deep features and hand-crafted morphometrics to build a more distinctive representation of neuronal morphology and improve the performance of neuron classification (Li et al., 2018; Zhang et al., 2021). They first employ two modules to acquire various features individually and then directly concatenate them to generate the final feature descriptors of neurons. For example, Li et al. (2018) first utilize Stacked Convolutional Auto-encoders (SCAEs) to extract deep features from three projected 2D images. Subsequently, the deep features are directly combined with hand-crafted morphometrics to generate a more distinct neuron representation. As illustrated in Figure 2A, Zhang et al. (2021) incorporate different features extracted from two data formats (i.e., SWC file and 2D slice images) to identify neuron types. Their work exploits a tree-based Recurrent Neural Network (RNN) and a CNN to extract morphological features from the SWC-format data and the 2D images, respectively. Then, these features are further concatenated, which shows power in the neuronal morphology representation and promotes the identification of neuron types. While these studies incorporate a variety of feature representations, they primarily rely on simple concatenation for fusion. It is inadequate to explore the shared information across different feature representations.

To effectively leverage the advantage of individual feature representation and the relationships and complementarity between different feature representations, we present a multi-level feature fusion network (as shown in Figure 2B). It effectively fuses hand-crafted morphometrics and deep features derived from 2D images by enhancing individual feature representation and mining the relationship and complementarity between different features. We support that the features extracted from hand-crafted morphometrics and 2D images are complementary. Deep features extracted from 2D images excel in capturing the holistic structure of neurons, while hand-crafted morphometrics provide precise statistical values for the local and global structures of neurons. Therefore, fusing these features significantly improves performance, as proved in Li et al. (2018) and Zhang et al. (2021).

To effectively merge these two features, the proposed multi-level feature fusion network aims to mine relationships and complementary information between different feature representations and facilitate their interaction. Specifically, our method first uses a Fully Connected Network (FCN) and a CNN to extract morphological characteristics from hand-crafted morphometrics and 2D images, respectively. Subsequently, we develop Multi-Level Fusion Modules (MLFMs) to enhance distinctive morphological information within each network and extract salient features across networks. The MLFM consists of a channel attention-based Feature Enhancement Module (FEM) and a cross-attention-based Feature Interaction Module (FIM). The FEM mines the discriminative information of each feature representation to capture a reliable depiction of neuronal shapes. Concurrently, the FIM mines the relationships and facilitates the exchange of information across both networks, thereby enriching the overall representation of neuronal morphology. Furthermore, MLFMs are embedded in multiple feature extraction blocks for more comprehensive learning of neuronal morphology. Finally, we further concatenate the feature representations extracted from FCN and CNN to effectively characterize neuronal morphology from both local and holistic levels. Experimental results demonstrate that our method can effectively capture the discriminative descriptors for neuronal morphology. Our method achieves an accuracy of 95.18% on the NeuroMorpho-10 dataset, outperforming that only based on individual feature representation.

The main contributions of our approach are listed:

- We develop a multi-level feature fusion network for neuron classification. This network fully characterizes neuron morphology by leveraging different feature representations and tactfully integrating them for effective neuron identification.
- The multi-level fusion modules are developed and embedded as bridges for information communication between two networks. It explores the distinguishable information of two features at multiple levels and then conveys it between the two networks to better characterize neuronal morphology.
- Our method accurately identifies 10 types of neurons with a high accuracy of 95.18%, superior to other methods by a large margin. Furthermore, our method performs well on the other two datasets, possessing good generalization.

The remainder of this article is structured as follows. Section 2 briefly introduces the related work. Section 3 elaborates the presented method, including data preprocessing, multi-level fusion network, and multi-level fusion module. Section 4 introduces the dataset, experimental settings, and experimental results and analysis. Section 5 discusses the model complexity, limitation, and improvement strategy of our proposed method. Finally, conclusions are presented in Section 6.

## 2 Related work

This section briefly introduces some related methods, including hand-crafted morphometrics, deep features-based neuronal morphology classification, and attention models.

### 2.1 Hand-crafted morphometrics-based neuron classification

To quantitatively characterize neuronal morphology, various hand-crafted morphometrics (Uylings and Van Pelt, 2002; Costa et al., 2010; Wan et al., 2015; Bird and Cuntz, 2019) are designed and widely utilized. These morphometrics can be primarily divided into four categories. They are distance-related metrics (e.g., branch length and Euclidean distance), angle-related metrics (such as bifurcation angles), topology-related metrics (including the number of bifurcations and branch order), and size-related metrics (like branch radius and surface area) (Chen et al., 2022). Typically, these features are summarized using statistics, such as mean or standard deviation. They are easily acquired using toolboxes (e.g., L-Measure Scorcioni et al., 2008, Neurolucida Glaser and Glaser, 1990, and nGauge Walker et al., 2021).

Traditional methods for neuronal morphology classification mainly rely on these hand-crafted morphometrics (Vasques et al., 2016; Laturnus et al., 2020; Cervantes et al., 2019). For example, 43 morphological measurements computed by the L-Measure toolbox (Scorcioni et al., 2008) are utilized to describe the characteristics of rat neurons (Vasques et al., 2016). Although these methods effectively identify neuronal morphology, they would fail to describe neurons with complex dendrites (Scorcioni et al., 2008). Other methods utilize the Sholl analysis (Sarkar et al., 2013; Khalil et al., 2022) and TMD (Kanari et al., 2018) to classify or cluster neurons. For example, Kanari et al. (2019) use TMD to classify cortical pyramidal cells in rat somatosensory cortex. Khalil et al. (2022) treat the multiple topological or spatial features of neurons as a function of distance from the soma and builds new Sholl descriptors. Their single descriptors perform better than traditional hand-crafted morphometrics in separating specific neuron types. More importantly, their results prove that combined descriptors can produce better classification or clustering performance than single descriptors. This motivates us to explore the fusion of multiple features for better classification or clustering for neuronal morphology.

### 2.2 Deep features-based neuron classification

Inspired by the advancements in deep learning techniques, some studies utilize CNNs to extract deep features from the 3D neuron data or 2D images (Lin and Zheng, 2018; Li et al., 2018; Zhang et al., 2021; Sun et al., 2023; Li et al., 2021; Zhao et al., 2022; Kanari et al., 2024). These methods directly feed 3D neuron data or 2D images into CNNs to automatically learn deep feature representation. Importantly, compared to the hand-crafted morphometrics, deep features obtained through CNNs are good at characterizing holistic structures of neurons (Li et al., 2018; Zhang et al., 2021). However, there are many difficulties in extracting deep features via CNNs directly from 3D neuron data. Specifically, the number of points in 3D space for neuron data varies greatly among various neuron samples. Consequently, creating a general neural network to extract deep characteristics from 3D neuron data is challenging. Additionally, if 3D neuron data is projected into 2D images, neuronal morphology information inevitably is lost during the projection process. As a result, the performance of these 2D projection-based approaches is constrained. Recent researches (Li et al., 2018; Zhang et al., 2021) attempt to create a feature vector by directly concatenating different features. While their results demonstrate that the fusion of diverse feature representations leads to performance gains, these improvements are constrained. This is because they do not consider the complementary and redundant information among the different features. Therefore, our method aims to leverage the strengths of these features and effectively fuse them to provide a precise characterization of neuronal morphology.

### 2.3 Attention model

Attention mechanisms discern and emphasize pivotal local regions, building more discriminative and pertinent features. Attention mechanisms have showcased remarkable success across various applications, such as image classification (Hu et al., 2018; Woo et al., 2018; Park et al., 2018; Hassanin et al., 2024), machine translation (Gehring et al., 2017; Vaswani et al., 2017), and visual question answering (Yu et al., 2017; Gao et al., 2018). For instance, in the domain of image classification, SENet (Hu et al., 2018) introduces a channel attention block to augment the representational capacity of neural networks. Woo et al. (2018) further extend these advancements by integrating channel and spatial attention modules into a unified block structure. Later, Vaswani et al. (2017) achieve another significant advancement in attention-based models based on the self-attention mechanism. The self-attention captures long-range dependencies effectively and provides interpretability by highlighting the relevance of different input components (Vaswani et al., 2017; Lin et al., 2017). It is highly scalable, parallelizable, and can be easily stacked in layers for more complex modeling. Here, our approach leverages a variant of self-attention, referred to as the cross-attention module, to effectively harness the dependencies and relationships of different features. This aims to facilitate the interaction of features extracted from different branches, promoting a more cohesive and integrated feature representation.

## 3 Methodology

**Figure 3:**
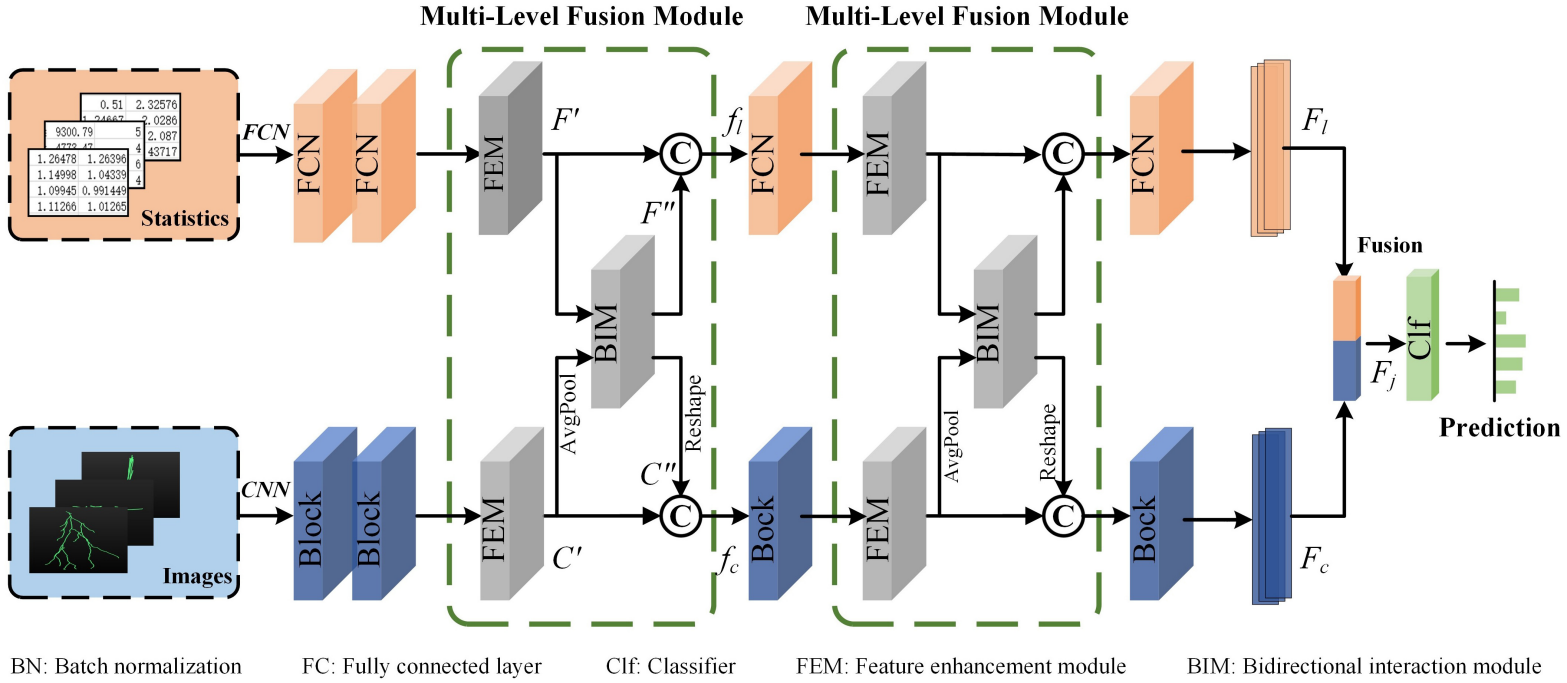
The proposed multi-level feature fusion network. An FCN is applied to extract geometric information from hand-crafted morphometrics. A CNN is utilized to characterize the holistic structure of neurons. The Multi-Level Fusion Module (MLFM), consisting of a Feature Enhancement Module (FEM) and a Feature Interaction Module (FIM), is embedded between the adjacent feature extraction blocks for information communication. Finally, the features (*F*_*l*_ and *F*_*c*_) extracted from the FCN and CNN are further fused to obtain a more distinguishing descriptor (*F*_*j*_).

The proposed multi-level feature fusion network dexterously integrates the morphological information derived from hand-crafted morphometrics and 2D images (as presented in Figure 3). It enhances the feature presentation within each branch while establishing connections between disparate feature representations to convey information effectively. Therefore, our method can achieve more accurate representation learning by taking full advantage of different feature representations and distinguishing information between them. Our multi-level feature fusion network mainly consists of two branches. Specifically, one branch utilizes an FCN to extract local geometric information from hand-crafted morphometrics. The other branch employs a CNN to capture deep features from the projected 2D images. The extracted deep features offer a more comprehensive characterization of the holistic structure of neurons. Moreover, the MLFM is embedded behind multiple feature extraction blocks for information enhancement and communication. MLFM first thoroughly mines the discriminative information of each feature representation using the channel attention-based FEM. Then, it discovers and transmits the interacted information between the FCN and CNN branches via cross-attention-based FIM. In this way, these two branches can comprehensively learn the morphological features of neurons. Finally, features extracted from these two branches are further fused to build a more distinguishing feature descriptor. The fused descriptor effectively reveals the differences and similarities among various neuron types.

### 3.1 Data preprocessing

In our work, the hand-crafted morphometrics and 2D images are taken into account simultaneously to learn a more distinguishing representation of neuronal morphology. Hand-crafted morphometrics almost measure the local geometry information of neurons, while deep features extracted from 2D projected images better characterize the holistic structures of neurons (Li et al., 2018).

There are many predefined feature measurements to measure neuronal dendrites and axons (Laturnus et al., 2020; Uylings and Van Pelt, 2002). Based on the neuron data stored in the SWC file, morphological features computed by the L-Measure toolbox (Scorcioni et al., 2008) are widely used to characterize morphologies of neurons quantitatively. They consist of total branch length, average branch diameter, average depth, number of bifurcations, maximum branch order, etc. As done in (Lin et al., 2018; Lin and Zheng, 2019; Li et al., 2018), we calculate 43 hand-crafted morphometrics (Figure 1D) using the L-Measure toolbox (Scorcioni et al., 2008) to measure the morphological characteristics of neurons comprehensively.

**Figure 4:**
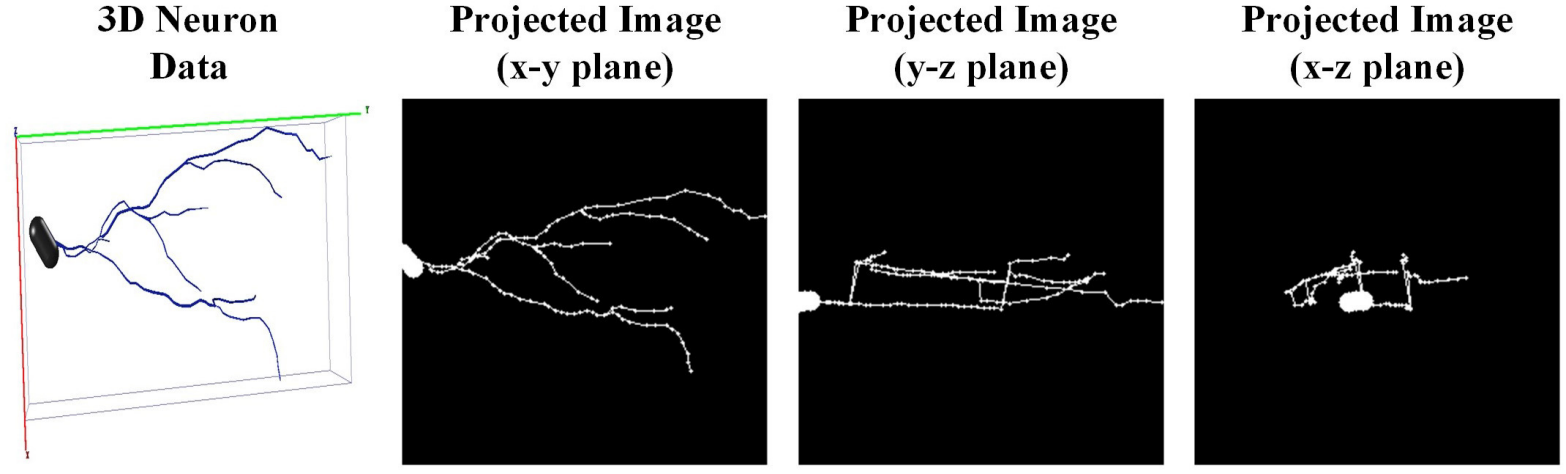
The projected 2D images of one granule cell. One 3D neuron data (the left one) is projected orthogonally into three angles of view (i.e., x-y, y-z, and x-z planes).

Designing a generalized 3D network to directly process 3D neuron data is challenging. This is due to the inherent characteristic of neuron reconstructions (e.g., relative sparse in 3D space, dramatic difference in the number of points). Therefore, we first project the 3D neuron data into 2D images as done in Li et al. (2018), Li et al. (2021), and Sun et al. (2023) and then utilize a 2D CNN to extract morphological features. We first extract the coordinates of neuron points and employ principal component analysis to normalize neurons into a normalized axis. Such an operation ensures the axis is consistent across neurons, without considering the origin axis provided by multiple laboratories. Then, to reduce the loss of morphological information, the 3D neuron data is projected orthogonally into three angles of view (i.e., x-y, y-z, and x-z planes). Thus, three projected images are obtained to fully describe different neuronal morphology (shown in Figure 4). Subsequently, these three projected images are stacked together along the channel and fed into CNN.

### 3.2 Multi-level fusion network

Given a 3D digital neuron reconstruction, we first preprocess it to obtain the hand-crafted morphometrics and 2D projected images. Subsequently, an FCN branch and a CNN branch are applied to extract neuronal morphological features from these two data formats. When the information flows through the two branches, the MLFMs we developed are embedded into the feature extraction block for information enhancement and communication. Besides, each branch can utilize the information from the other to facilitate their representation learning.

For the FCN branch, considering that the hand-crafted morphometrics are statistical summary values and the number of them is limited, we opt to employ a shallow FCN to extract features. It consists of several FCN blocks. Note that the number of FCN blocks is consistent with the number of blocks in the CNN for better information communication. Besides, each FCN block includes three Fully Connected (FC) layers, two Batch Normalization (BN) layers, and two ReLU (Nair and Hinton, 2010) layers. The dimensions of the output of the first two FC layers are half and a quarter of the dimension of the input, respectively. To better perform information exchange channel-wise between two networks in the MLFM, the dimension of the output of the third FC layer is set to be the same as the number of channels of the corresponding convolution block in the CNN.

For the CNN branch, we first stack three projected images together along the channel and then feed them into CNN to extract deep features. Here, we set the CNN as the ResNet-50 (He et al., 2016). Considering that the output of the first block is relatively specific, information communication with the FCN occurs starting from the second convolution block. Therefore, three MLFMs are added to bridge the information communication between the FCN and CNN. Each MLFM receives the features extracted from the FCN and CNN. Then, it enhances each feature through the channel attention-based FEM. Next, it discovers and transmits the interacted information between the FCN and CNN branches via cross-attention-based FIM. In this way, these two branches can comprehensively learn the morphological features of neurons with the help of the other branch.

To achieve a more comprehensive characterization of neuronal morphology, we further fuse features extracted from the FCN and CNN branches to generate a more informative vector as the final descriptor (denoted as *F*_*j*_). Subsequently, a classifier, consisting of two FC layers, is employed to categorize neurons based on this final descriptor. The dimensions of the output of these FC layers are the dimension of the lasted fused feature and the number of neuron types, respectively.

### 3.3 Multi-level fusion module

To fully capture the neuronal morphology, some approaches concatenate different feature vectors into a vector directly (Li et al., 2018; Zhang et al., 2021). However, these methods do not thoroughly consider the complementarity of different features. To capture informative features for each branch and the complementarity between different branches, we introduce the MLFM. It includes a channel attention-based FEM and a cross-attention-based FIM.

**Figure 5:**
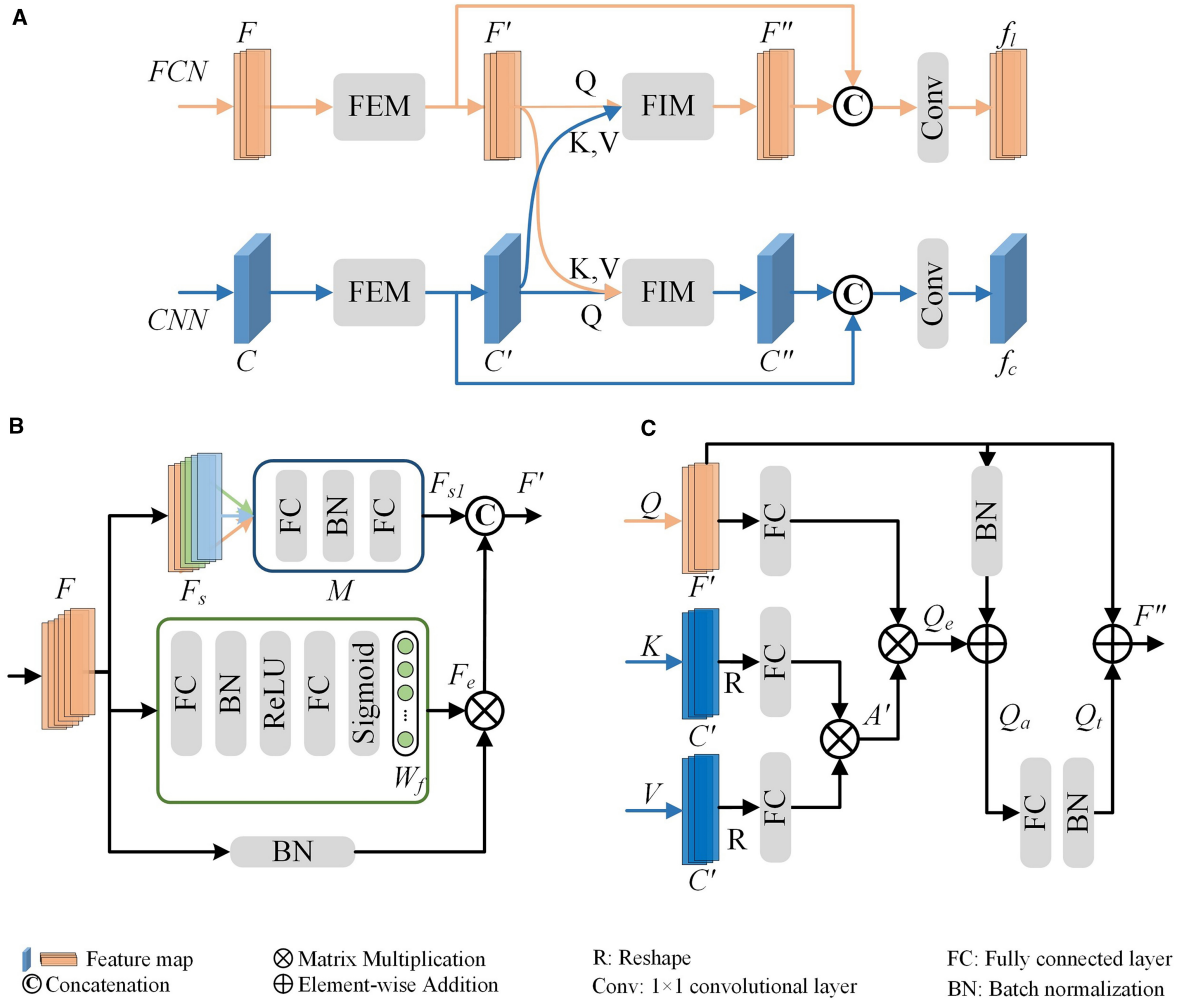
Multi-Level Feature Fusion Module (MLFM) **(A)**. It mainly consists of the channel attention-based Feature Enhancement Module (FEM) **(B)** and the cross-attention-based Feature Interaction Module (FIM) **(C)**. The FEM is first utilized to obtain the enhanced features within each branch (*F*′ for the FCN branch or *C*′ for the CNN branch) and then the FIM is used to mine the relationships and shared information between two branches. To fully use the features of each branch and the shared features, the MLFM integrates them to produce a more distinguishing feature (*f*_*l*_ or *f*_*c*_).

As presented in Figure 5A, the MLFM takes the output (denoted as *F* and *C*) of the feature extraction block in the FCN and CNN branch as the input. It first utilizes the channel attention-based FEM in each branch to capture the salient features (*F*′ and *C*′). Then, the salient features are fed to cross-attention-based FIM to mine the complementary features between *F*′ and *C*′. Finally, the outputs of these two modules are concatenated to generate a more informative feature (i.e., *f*_*l*_ and *f*_*c*_ for FCN and CNN branches, respectively). The fused feature is then fed into the next feature extraction block to facilitate the presentation learning of each branch. To keep the dimension of the concatenated features as same as the input (*F*), an FC layer in the FCN branch is employed to reduce the dimension of the fused features. Similarly, a convolution layer with a kernel size of 1 × 1 in the CNN branch is utilized to reduce the feature dimension. Therefore, the concatenated features can be directly fed back into the FCN and CNN branches to further convey information in the next feature extraction block.

**Feature Enhancement Module (FEM)**. The FEM mines salient features within a single branch to dynamically enhance and update reliable information. Here, we illustrate the FEM using the FCN branch as a representative example (as shown in Figure 5B).

To enhance the discriminability of features, especially given the sparseness of neurons, we develop a channel attention-based FEM. As depicted in the green box of Figure 5B, the FEM first calculates the weights (*W*_*f*_) for *F*. This is achieved through two FC layers and a sigmoid function as follows,


(1) $$\begin{array}{l} W_{f}=\sigma \left(fc\left(\delta \left(BN\left(fc\left(F\right)\right)\right)\right)\right), \end{array}$$

where δ is the ReLU function (Nair and Hinton, 2010), *fc* is the FC layer, and σ is the sigmoid function. The feature dimensions of FC layers are half and full of the dimension of *F*, respectively. The sigmoid layer σ enables our module to learn non-linear interactions between channel features. It allows for the emphasis of multiple channel features. The enhanced features *F*_*e*_ are then defined by applying the calculated weights to the normalized features as,


(2) $$\begin{array}{l} F_{e}=W_{f}*BN\left(F\right). \end{array}$$

Such operations ensure that the FEM effectively improves the discriminative power of features by leveraging channel attention mechanisms.

Furthermore, to address the morphological diversity of neuronal arbors, we implement a group-wise refinement process for the global features *F*. Specifically, we divide the global features (*F*) into *N*_*s*_ parts along the channels, resulting in grouped features (*F*_*s*_). As illustrated in Figure 5B, the green, orange, and light blue rectangles represent different grouped features. Drawing from the optimal outcomes of our previous research, we set *N*_*s*_ as 3. For each group, we apply a feature learning module *M* to enhance the grouped feature. The *M* consists of two FC layers and a BN layer. The enhanced grouped features *F*_*s*1_ are then constructed by concatenating the outputs of the module *M* applied to each group as follows:


(3) $$\begin{array}{l} F_{s1}=\left[M\left(F_{s_{0}}\right),M\left(F_{s_{1}}\right),\cdots \;,M\left(F_{s_{N_{s}}}\right)\right], \end{array}$$

where [·] is the concatenation operation. Subsequently, the final enhanced global features (*F*′) are generated by concatenating the previously enhanced features *F*_*e*_ with the newly enhanced grouped features *F*_*s*1_. Besides, an FC layer is utilized to reduce the dimension of the *F*′ as the same as *F*.

For the CNN branch, the same operation is conducted to capture the final enhanced features (*C*′). Note that the feature (*C*) is first fed to one average pooling layer to get global features and then fed into the FEM to extract enhanced features. Moreover, a 1 × 1 convolution layer is utilized to reduce the dimension of the *C*′ as the same as *C*.

**Feature Interaction Module (FIM)**. The proposed FIM (as shown in Figure 5C) is designed to facilitate dynamic interaction between the two representations. It facilitates our model to effectively extract more distinctive and comprehensive morphological representations for neuronal morphology.

We obtain the enhanced features *F*′ and *C*′ through FEM for the FCN and CNN branches, respectively. The FIM first transforms them into one-dimensional feature vectors with the same dimension. This can be achieved through average pooling or reshaping operations. We leverage the principles of the cross-attention mechanism (Galassi et al., 2020; Woo et al., 2018; Park et al., 2018) to uncover the relationships and shared information between different branches, thereby enhancing the feature learning for each branch. In this process, features from one branch act as the query (*Q*), while those from the other branch serve as the key (*K*) and value (*V*). The shared features between the FCN and CNN branches are obtained through a scaled dot-product operation, as delineated as:


(4) $$\begin{array}{l} \begin{array}{c} Q_{e}=softmax\left(\frac{QK^{T}}{\sqrt{d}}\right)V, \end{array} \end{array}$$

where *d* is the channel dimension of features. The captured shared features are crucial for promoting the mutual refinement of different branches.

To amplify the distinctive traits of the FCN branch, we fully utilize the input *Q* (i.e., *F*′) to generate the final interactive feature *F*″. This involves a multi-step process, including the application of an addition layer, an FC layer, and a BN layer, as expressed as:


(5) $$\begin{array}{l} \begin{array}{c} F^{\prime \prime }=BN\left(fc\left(BN\left(F^{\prime }\right)+Q_{e}\right)\right)+F^{\prime }, \end{array} \end{array}$$

where *fc* represents the FC layer, and BN signifies batch normalization. Similarly, we can obtain *C*″ for the CNN branch through the same operations. In this way, the FIM is meticulously designed to fortify the branch's distinctiveness by integrating the adaptive attention features and fostering collaborative learning between the two branches.

### 3.4 Loss function

The designed multi-level feature fusion network comprehensively characterizes neuronal morphology by fully improving the feature learning of FCN or CNN and tactfully integrating different features. Therefore, our learning goal is to train the CNN and FCN to extract distinguishable characteristics and promote the accurate classification of neurons. The objective function is:


(6) $$\begin{array}{l} L=-\frac{1}{N}\overset{N}{\underset{i=1}{\sum }}\overset{c}{\underset{j=1}{\sum }}y_{ij}log\left(p_{ij}\right), \end{array}$$

where *N* is the number of samples, *c* is the number of classes, and *y*_*ij*_ is the true neuron type provided by experts. As shown in Figure 2, the *p*_*ij*_ is the prediction given by the classifier based on the fused features *F*_*j*_, where *F*_*j*_ are derived from the features *F*_*l*_ and *F*_*c*_ extracted from the FCN and CNN branches.

## 4 Experimental results

In this part, we first introduce the validation dataset and experimental settings. Then we present the performance of our method and the comparison results with other methods. Next, we verify the generalization of our approach. Finally, we perform multiple ablation studies to specifically analyze the performances of our modules.

### 4.1 Datasets and implementation details

#### 4.1.1 Dataset

**Figure 6:**
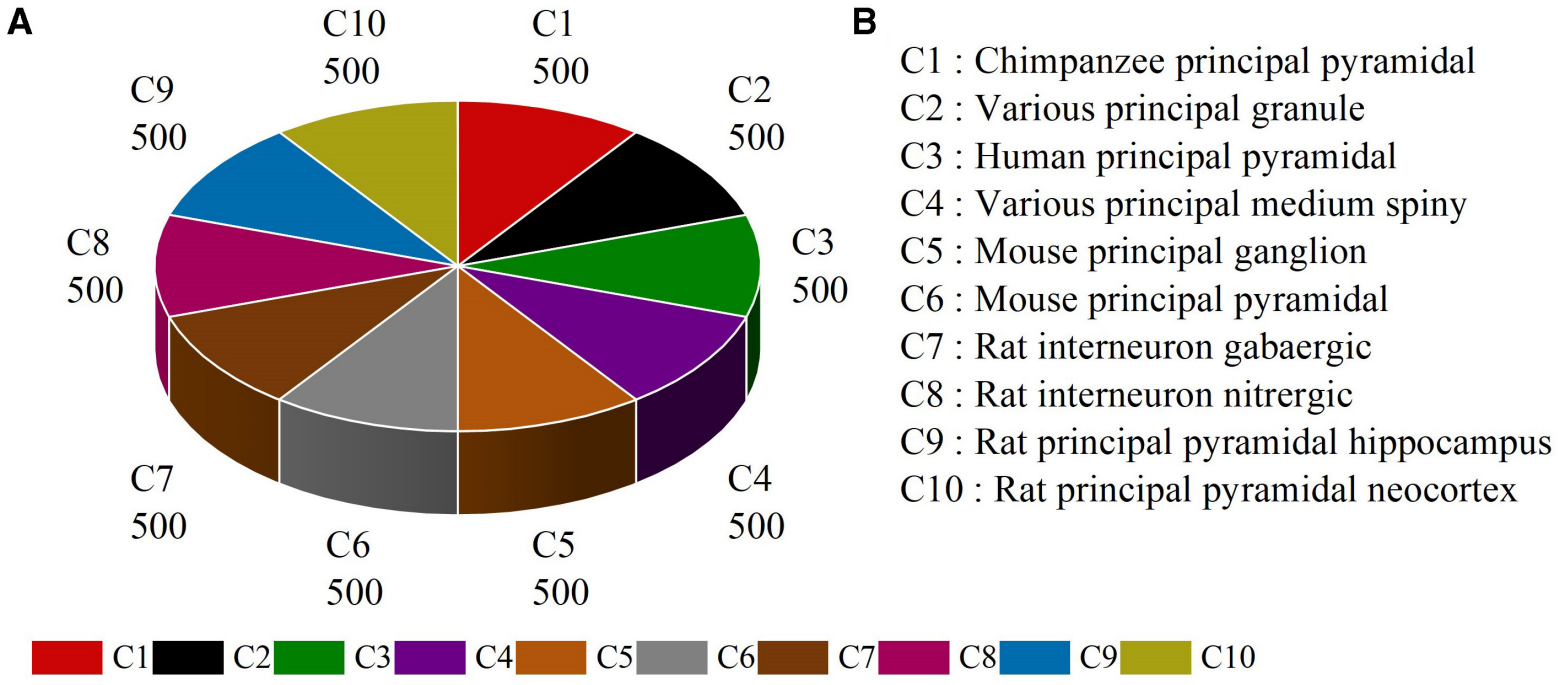
The class distribution **(A)** and class name **(B)** of the NeuroMorpho-10 dataset.

Several types of neurons from different brain regions and species can be found in publicly available NeuroMorpho.org (Ascoli et al., 2007). Here, neurons belonging to different species and brain regions are selected and downloaded from NeuroMorpho.org (Ascoli et al., 2007) to evaluate the effectiveness of our multi-level feature fusion network. To make a fair comparison, we utilize the same neuron types reported by the work (Cervantes et al., 2019). This dataset includes 5,000 neurons from 10 types, denoted as NeuroMorpho-10. As shown in Figure 6, the NeuroMorpho-10 dataset includes chimpanzee pyramidal cells, granule cells, human pyramidal cells, medium spiny, mouse ganglion cells, mouse pyramidal cells, rat GABAergic, and nitrergic interneurons, and rat pyramidal cells in the hippocampus and neocortex. For clarity, they are denoted as C1, C2, C3, C4, C5, C6, C7, C8, C9, and C10, respectively. For each class, there are 500 digital reconstructions of neurons. All data can be found in our Google drive.^1^

#### 4.1.2 Implementation details

The multi-level feature fusion network is implemented in PyTorch and trained on two NVIDIA GTX 2080Ti GPU cards. For the CNN branch, all projected images are obtained and resized to 224 × 224. For the FCN branch, 43 hand-crafted morphometrics are computed by L-measure tool (Scorcioni et al., 2008) as the input of the FCN. The weights of CNN are initialized with the pre-trained weights on ImageNet. Our preliminary results find that the CNN initialized with pre-trained weights outperforms that is trained from scratch. Furthermore, batch normalization is applied to every hidden layer. Dropout with a ratio of 0.5 is applied after the FC layers of the classifier. We use an Adam (Kingma and Ba, 2014) optimizer with an initial learning rate of 1e-3 with cosine learning rate decay to optimize our multi-level feature fusion network. A total epoch of 100 is conducted with a mini-batch size of 16.

#### 4.1.3 Evaluation metrics

The average overall accuracy of 10-fold cross-validation is exploited to validate the performance of the proposed network. Additionally, the precision, recall, and F1-score are employed to measure the efficiency of our method. Furthermore, the Receiver Operating Characteristic (ROC) curves and the Area Under the Curve (AUC) are utilized to evaluate our model. Moreover, we provide the feature distribution plots computed by the t-SNE tool (Van der Maaten and Hinton, 2008) and confusion matrix to clearly show the performance of our multi-level feature fusion network.

### 4.2 Performance of our method

The proposed multi-level feature fusion network mines the salient information from 2D images and hand-crafted morphometrics from multiple levels. It conveys fused and complementary features to CNN and FCN to enhance their representation learning. We first verified the performance of the CNN and FCN based on the images and hand-crafted morphometrics, respectively. As illustrated in Table 1, the classification accuracy of CNN with images as input can reach 85.44%. The FCN with geometric statistics as input can identify 10 types of neurons with an accuracy of 82.40%. The proposed multi-level feature fusion network makes full use of features obtained from these two inputs. It enhances the distinguishing features from one network and propagates the distinguishing information across these two networks on multiple levels. Therefore, our method reaches an accuracy of 95.18%.

**Table 1:** Performance comparison of different methods based on different feature representations.

**Method**	**Precision**	**Recall**	**F1-score**	**Accuracy (%)**
CNN	0.855	0.854	0.854	85.44
FCN	0.827	0.824	0.820	82.40
Ours	0.952	0.952	0.952	95.18

**Figure 7:**
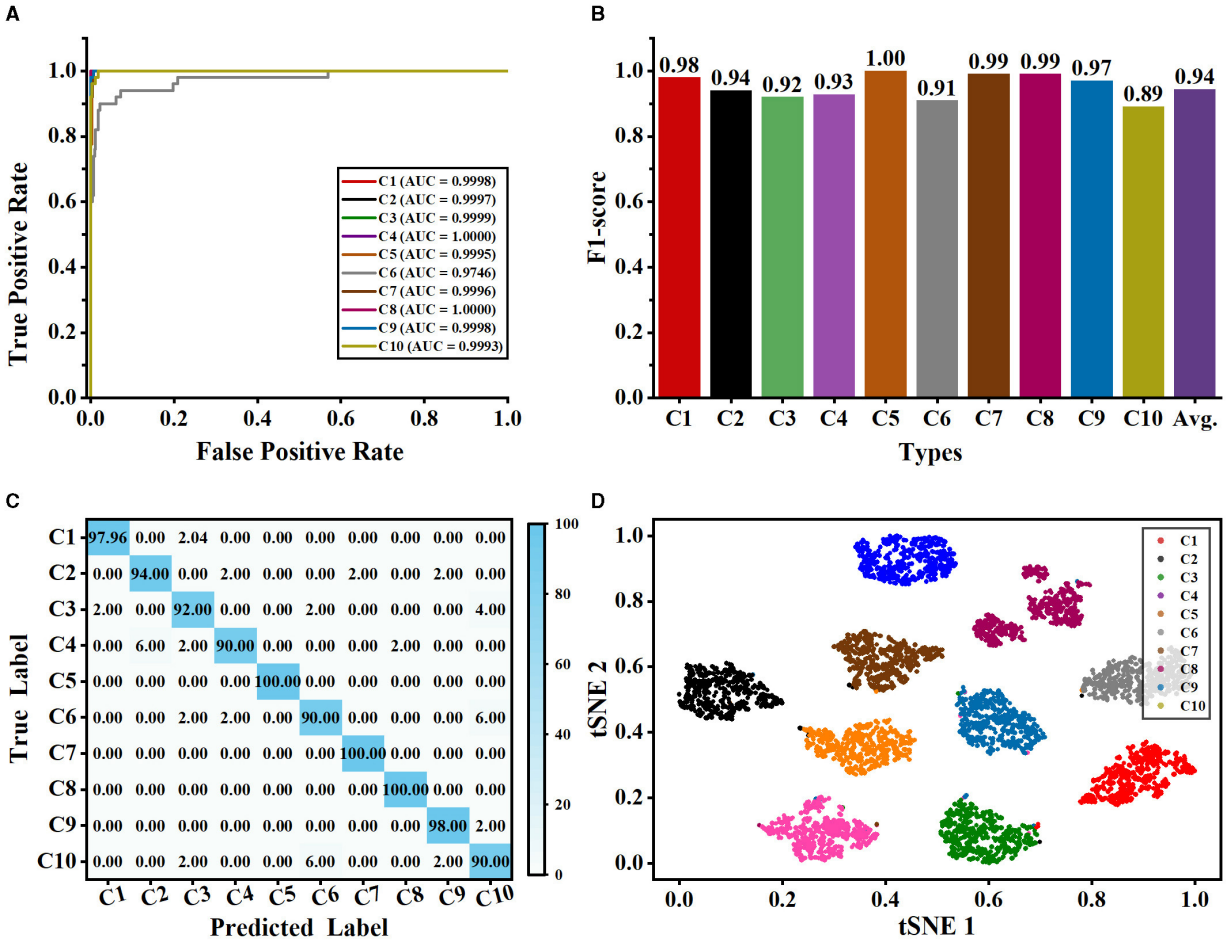
Classification performance of our method evaluated on the NeuronMorpho-10 dataset with 10-type neurons. **(A)** is the ROC curves of our method for each type. **(B)** is the F1-score for each type. **(C)** is the confusion matrix. **(D)** is the feature distribution.

As shown in Figure 7, our method can accurately and reliably identify each type of neuron (as shown in Figures 7A, B). Although different types of neurons share similarities in their shape and size, the proposed multi-level fusion network is still able to effectively capture the differences between types and represent them accurately. Our method achieves excellent AUC and F1-score for each type of neuron. Specifically, our method achieves satisfactory AUC values for each type, where AUC values exceed 0.999 for most classes. Furthermore, our method achieves the AUC values of 1.000 for C4 and C8, indicating our method can be used to precisely analyze the neurons from C4 and C8 types. Moreover, our method yields an average F1-score of 0.94 and the F1-scores of all classes exceed 0.89. This demonstrates that our method effectively represents the differences among neuron classes and accurately identifies neurons. Most of the misclassified samples by our method are from C6 (i.e., mouse pyramidal cells) and C10 types (i.e., mouse pyramidal cells) (as shown in Figure 7C). Specifically, 6% of neurons from the C6 type are misclassified as C10 type, while 6% of neurons from the C10 type are misclassified as C6 type (as shown in Figure 7C). This is because there are some morphological similarities between these two types. For example, the average contraction of neurons from C6 and C10 are 0.8949 ± 0.0571 and 0.8925 ± 0.0611, respectively. The ratio between the diameter of a daughter and its father of neurons from C6 and C10 are 0.8642 ± 0.1733 and 0.8502 ± 0.0965, respectively. This indicates that our method should be further modified to effectively learn and identify these subtle morphological nuances. Therefore, our future work will focus on improving the ability of our method to represent subtle differences so that it can accurately describe the subtle morphological properties of neurons.

Besides, the t-SNE tool (Van der Maaten and Hinton, 2008) is exploited to visualize the feature distributions of the last layer of our network. As seen from Figure 7D, each category is grouped into a cluster and neurons in each cluster are relatively compact. This indicates that our method can effectively learn and characterize the morphological features of each type of neuron. Besides, we observe that the neurons in the C4 cluster (i.e., various principal medium spiny) distribute relatively sparse compared to other clusters. We randomly select the neurons of the C4 type without considering their species and brain regions. Moreover, neurons in the C4 type have more dendritic spines than other cells. These factors may cause relatively large morphological differences among neurons in the C4 type. Nonetheless, our method accurately characterizes the morphological differences of neurons in the C4 type and groups them. Furthermore, there is a great deal of distance between different clusters. These results further prove that our method can precisely different types of neurons and fully using different features is conducive to recognizing neuron types.

### 4.3 Comparison of different methods

These neuron classification methods based on hand-crafted morphometrics and traditional classifiers are re-implemented to make a comparison with our method, including TMD (Kanari et al., 2018), novel Sholl analysis (Khalil et al., 2022), Support Vector Machine (SVM; Lee et al., 2004), Naïve Bayes (NB; Mihaljević et al., 2015), Linear Regression (LR; Alavi et al., 2009), and k-Nearest Neighbors (KNN; Wu et al., 2008). The recent methods that take hand-crafted morphometrics as input and neural networks as feature extractors are also compared, including Deep Residual Neural Networks (DRNN; Lin et al., 2018) and Locally Cumulative Connected Deep Neural Networks (LCCDNN; Lin and Zheng, 2019). The MorphoGNN (Zhu et al., 2022) method employs Graph Neural Networks (GNN) to learn the spatial structure information between the nodes of reconstructed neuron fibers. Besides, the SCAEs and TRNN+CNN extract features from 2D neuron images and hand-crafted morphometrics or SWC data are taken as a comparison (Zhang et al., 2021; Li et al., 2018). They concatenate different features directly to generate a feature descriptor. Additionally, we compare our method to the methods (Lin and Zheng, 2018; Chen et al., 2022) directly taking the 3D neuron data as input. Table 2 reports the classification performance of different methods.

**Table 2:** Performance comparison of different methods.

**Method**	**Data input**	**Feature**	**Precision**	**Recall**	**F1-score**	**Accuracy (%)**
SVM (Lee et al., 2004)	Statistics	Hand-crafted	0.561	0.537	0.487	53.66
NB (Mihaljević et al., 2015)	Statistics	Hand-crafted	0.614	0.651	0.588	65.06
LR (Alavi et al., 2009)	Statistics	Hand-crafted	0.770	0.769	0.755	76.88
KNN (Wu et al., 2008)	Statistics	Hand-crafted	0.817	0.813	0.803	81.28
TMD (Kanari et al., 2018)	3D Neuron Data	Hand-crafted	0.754	0.803	0.629	80.27
Sholl (Khalil et al., 2022)	3D Neuron Data	Hand-crafted	0.923	0.901	0.900	90.00
DRNN (Lin et al., 2018)	Statistics	Deep Feature	0.595	0.540	0.502	54.00
LCCDNN (Lin and Zheng, 2019)	Statistics	Deep Feature	0.604	0.552	0.534	55.20
3D CNN (Lin and Zheng, 2018)	3D Neuron Data	Deep Feature	0.439	0.458	0.429	45.82
TreeMoco (Chen et al., 2022)	3D Neuron Data	Deep Feature	0.756	0.757	0.753	75.70
MorphoGNN (Zhu et al., 2022)	3D Neuron Data	Deep Feature	0.859	0.858	0.858	85.89
TRNN + CNN (Zhang et al., 2021)	3D Neuron Data + Images	Deep Feature	0.838	0.850	0.836	85.00
SCAES (Li et al., 2018)	Statistics + Images	Deep Feature	0.622	0.628	0.623	62.76
Ours	Statistics + Images	Deep Feature	**0.952**	**0.952**	**0.952**	**95.18**

The best results are shown in bold.

As seen from Table 2, the classification performance of these methods that only use hand-crafted morphometrics to characterize neurons is barely satisfactory, and the highest accuracy is only 81.28% achieved by the KNN method (Wu et al., 2008). Since hand-crafted morphometrics are a set of statistical measurements and have 43 dimensions, they are not good at describing the holistic structures of neurons with complex morphological structures. Therefore, these methods perform relatively weaker. In contrast, our method characterizes neurons by integrating hand-crafted morphometrics with deep features extracted from 2D view images. This allows our method to capture a comprehensive descriptor for neuronal local and holistic structures. Consequently, our approach effectively identified neuronal types. The novel Sholl method built by Khail et al. achieves satisfactory performance with an accuracy of 90.00%. However, creating a tree structure to obtain the Sholl descriptors requires higher computation resources and time, especially for neurons with a larger number of points (such as those >8,000). While MorphoGNN achieves an accuracy of 85.89%, it requires processing the number of neuron points to a unified input size, such as 1,024 or 2,048. The larger the input size, the higher the computation resources. Note that the number of neuron points ranges from 10 to 10,000. Therefore, their method risks losing significant morphological information for neurons with greater points and introducing noise for neurons with fewer points. Although our method utilizes the same input as the SCAEs (Li et al., 2018), our method utilizes the MLFM to facilitate the information interaction between different inputs at multiple levels. Conversely, SCAEs directly connect deep features and hand-crafted morphometrics. Our method can obtain complementary information between different features and effectively use their advantages. Therefore, the performance of SCAEs is relatively lower than that of our method. Besides, the TRNN+CNN (Zhang et al., 2021) method utilizes CNN and tree-based RNN to capture morphological features from SWC format data and 2D slice format data. However, it directly fuses different features only before the classifier to build the morphological descriptors. Compared to the SCAEs (Li et al., 2018) and TRNN+CNN (Zhang et al., 2021), our method fully uses two different features and tactfully integrates them at multiple scales during feature extraction. Furthermore, it mines and shares complementary information between two features via the MLFM. Consequently, our method yields an accuracy of 95.18% and an F1-score of 0.952, outperforming other methods.

### 4.4 Evaluation of generalization ability

Here, we verify the generalization ability of our method from many aspects. In this section, we utilize the same data preprocessing described in Section 3.1 and experimental setting reported in Section 4.1.2 on different datasets to conduct the evaluation experiments. Researchers analyzing neuronal morphology can conveniently find the datasets on our Google drive (see text footnote ^1^).

**Figure 8:**
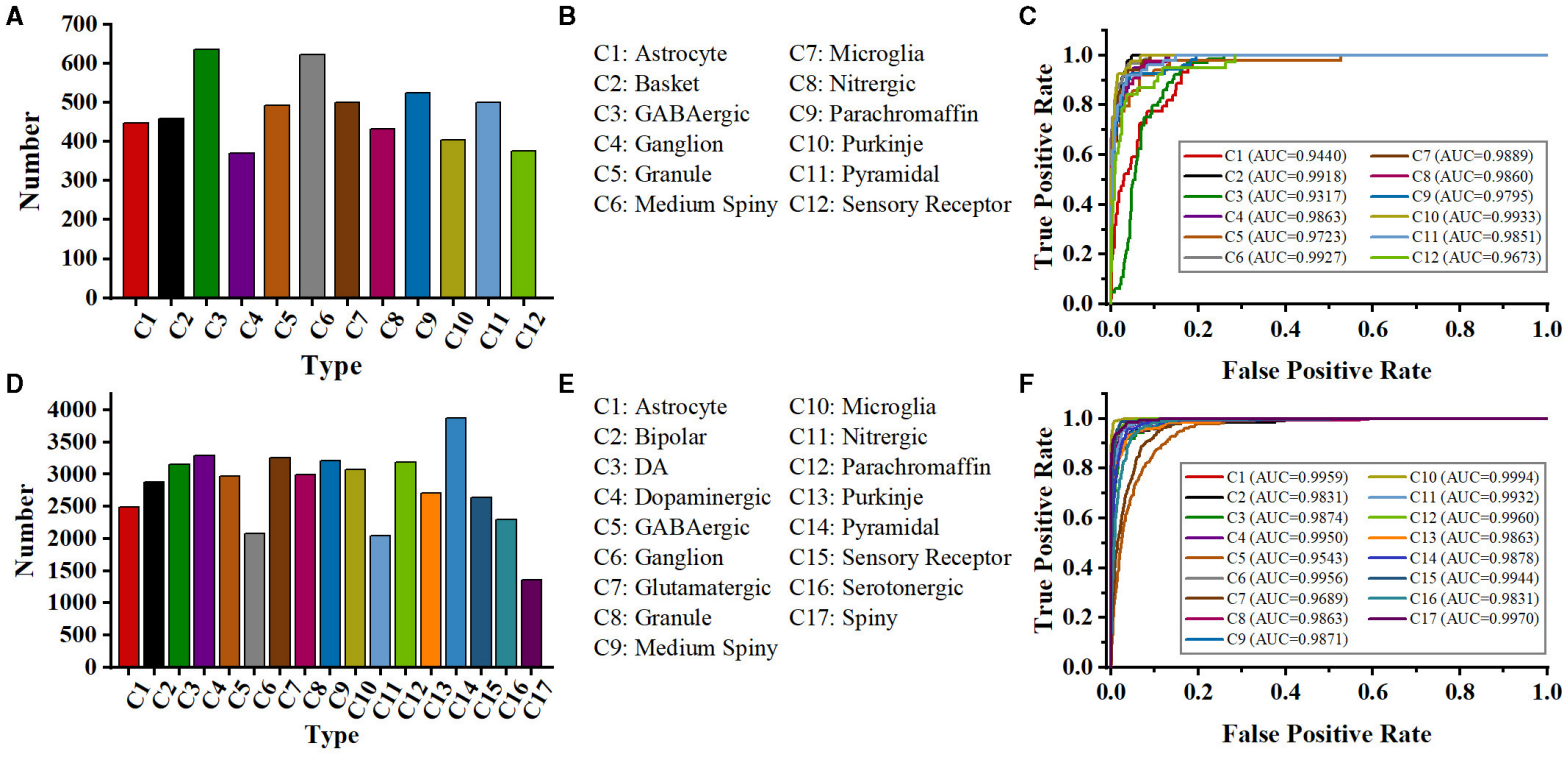
Class distribution of NeuroMorpho-12 and NeuroMorpho-17 datasets and ROC curves of our methods for each type. The 12 types of neurons **(A)** and type name **(B)** of the NeuroMorpho-12 dataset. **(C)** is the ROC curves of our method for 12 types of neurons in the NeuroMorpho-12 dataset, **(D, E)** are the 17 types of neurons and the name of the NeuroMorpho-17 dataset, respectively, **(F)** is the ROC curves of our method for each type in the NeuroMorpho-17 dataset.

In the work of Zhang et al. (2021), a tree-based RNN is used to process the SWC format data, and a CNN is employed to extract features from 2D slice images. To verify their method, they download 35,000 digital neuron reconstructions from NeuroMorpho.org (Ascoli et al., 2007). Moreover, they augment the training images to 98,700 and SWC-format data to 99,996. The model concatenates the features extracted by CNN and RNN and achieves an accuracy of 91.90%. The same dataset as that in their work is exploited to evaluate our proposed method (denoted as NeuroMorpho-12). NeuroMorpho-12 includes the 12 types of cells (as shown in Figures 8A, B). We use only 5,752 cells provided in their study (Zhang et al., 2021) and do not utilize data augmentation techniques. Figure 8C shows the ROC curves of our method for each type. Our method identifies 12 types of neurons with satisfying AUC values. See from Table 3, our method achieves a classification accuracy of 92.00%. Note that, our method makes comparable results with Zhang et al. (2021), but the number of neurons used in our method only is 1/19 of that in Zhang et al. (2021). Our multi-level fusion method not only enhances the individual feature representations but conveys the interacted information between different feature representations. Consequently, our method achieves comparable results with smaller neurons compared to that (Zhang et al., 2021) directly concatenating feature presentations.

**Table 3:** Validation of the generalization of our method evaluated on other two datasets.

**Dataset**	**Precision**	**Recall**	**F1-score**	**Accuracy (%)**
NeuroMorpho-12	0.922	0.920	0.919	92.00
NeuroMorpho-17	0.909	0.901	0.902	90.10

Besides, a dataset (denoted as NeuroMorpho-17) consisting of 17 classes of cells is utilized to verify the performance of the proposed method. The NeuroMorpho-17 consists of 47,460 cells downloaded from the NeuroMorpho.org (Ascoli et al., 2007). As shown in Figures 8D, E, this dataset includes more types compared to the NeuroMorpho-10 and NeuroMorpho-12 datasets. The number of neurons in each type ranges from 1,000 to 3,000, making neuron identification challenging. As shown in Figure 8F, our method yields excellent AUC values for each type. This indicates that our method fits well in identifying more types of neurons. Moreover, as shown in Table 3, our method yields an accuracy of 90.10% and an F1-score of 0.902, which is comparable to that of the other two datasets. Our method extracts morphological features of neurons from the hand-crafted morphometrics and 2D images and tactfully fuses them at the multi-level. It can more accurately characterize the structure of neurons and the differences between different neurons. Therefore, although neurons in this dataset have more complex arbors and exhibit more morphological diversity, our method still effectively characterizes their morphologies and accurately identifies them. More importantly, these results show the capability of our method to discern a broader range of neuron types and open up a novel avenue for the analysis of large-scale neuronal morphology.

### 4.5 Ablation studies

#### 4.5.1 Evaluation of different modules

In the MLFM, FEM aims to enhance feature learning within one network, and FIM aims to explore distinguishing and shared information between different features extracted from the CNN and FCN. Here, we verify the impact of these two modules on our model, and the results are shown in Table 4.

**Table 4:** Performance of our method with different modules.

**FEM**	**FIM**	**Precision**	**Recall**	**F1-score**	**Accuracy (%)**
-	-	0.877	0.878	0.877	87.78
✓	-	0.930	0.930	0.930	93.02
-	✓	0.903	0.904	0.904	90.35
✓	✓	0.952	0.952	0.952	95.18

We observe that the method that directly concatenates the features from the two networks achieves an accuracy of 87.78%. It has improvements of 2.34% and 5.38% than 85.44% and 82.40% only using CNN and FCN, respectively. This shows that the fused features can better represent the morphology of neurons and facilitate the classification of neurons. When the FEMs are introduced, each network can pay more attention to the discriminative features, which can further improve the classification performance. FIM enables CNN and FCN to make better use of the shared and transfer information between different feature representations. This improves the accuracy of our method to 90.35%. When the multi-layer fusion modules containing these two modules are embedded in the feature extraction block, our model can both enhance the salient features within the branch and fully mine the interaction information between different features. Therefore, our method achieves a satisfactory accuracy of 95.18%.

#### 4.5.2 Performance of our method with different fusion strategies

We employ element-wise summation to fuse the features from the CNN and FCN to obtain the descriptors of neurons. The fusion strategies, such as concatenation and average, are also commonly used to combine different features. Therefore, we evaluate our method based on different fusion strategies.

As shown in Table 5, our method based on the concatenation fusion strategy yields an accuracy of 93.57%. By applying the average fusion strategy to combine various features, our method improves accuracy to 94.78%. Our method based on the element-wise summation fusion strategy achieves an accuracy of 95.18%. Based on the MLFM, our method effectively utilizes different morphological features and characterizes neuronal morphology fully. Therefore, the feature descriptors generated through these fusion strategies capture the morphological properties of neurons more effectively. Different fusion strategies have minimal impact on accuracy compared to other modules. Consequently, we select the element-wise summation fusion strategy as our final fusion method due to its superior performance.

**Table 5:** Comparison of different fusion methods evaluated on 10 types of neurons.

**Method**	**Precision**	**Recall**	**F1-score**	**Accuracy (%)**
Concatenation	0.936	0.936	0.935	93.57
Average	0.950	0.948	0.947	94.78
Ours	0.952	0.952	0.952	95.18

#### 4.5.3 Performance of our method with different CNNs

Here, the performance of the multi-level feature fusion network using different CNNs is verified, and the results are shown in Table 6. When the VGG (Simonyan and Zisserman, 2014) network is employed to learn neuronal characteristics, the obtained feature maps cannot properly reflect the characteristics of neurons and even confuse neuron samples with very simple structures (e.g., neurons only with two arbors). On the other hand, when using ResNet (He et al., 2016) networks with a short connection structure, each layer's input and output are fully utilized so that the feature maps thoroughly reflect the structure and characteristics of neurons. Therefore, the identification of neurons is more accurate with the CNN based on ResNet (He et al., 2016) as the image feature extractor. The neuron data is relatively sparse, overfitting may occur when the deeper ResNet serves as the image feature extractor. Our method based on the ResNet-50 achieves the highest accuracy of 95.18% while that of ResNet-101 is 92.83%. Therefore, ResNet-50 is used as an image feature extractor in the CNN branch for more accurate representation learning.

**Table 6:** Comparison of the CNN branch of multi-level fusion network under different backbones.

**Backbone**	**Precision**	**Recall**	**F1-score**	**Accuracy (%)**
VGG-16	0.821	0.808	0.806	80.82
VGG-19	0.874	0.867	0.867	86.66
ResNet-18	0.909	0.903	0.904	90.34
ResNet-34	0.906	0.906	0.905	90.56
ResNet-50	0.952	0.952	0.952	95.18
ResNet-101	0.928	0.928	0.928	92.83

## 5 Discussions

Neuronal morphology varying in size and shape can result in challenges in accurately identifying neuron types. Previous studies employ both hand-crafted morphometrics (Uylings and Van Pelt, 2002; Costa et al., 2010; Vasques et al., 2016; Zehtabian et al., 2022; Wan et al., 2015; Bird and Cuntz, 2019) and deep features extracted by neural networks from 2D images (Li et al., 2018; Zhang et al., 2021; Li et al., 2021) to characterize neuronal morphology. The hand-crafted morphometrics provide statistical values for neuronal local and global structure. However, it often struggles to identify neurons with complex dendrites (Zhao et al., 2022; Chen et al., 2022). In contrast, deep features effectively capture the holistic structure of neurons (Li et al., 2018). However, converting 3D neuron data into 2D images results in a loss of structure information. It may lead to a decrease in classification performance. Our multi-level fusion network leverages the strengths of both hand-crafted and deep features and effectively combines them. Therefore, our approach enables the effective capture of discriminative descriptors for neuronal morphology. Furthermore, our method achieves superior performance on the NeuroMorpho-10, NeuroMorpho-12, and NeuroMorpho-17 datasets.

Our multi-level fusion network incorporates MLFMs to facilitate feature enhancement and interaction between different features. Each MLFM consists of a FEM and a FIM. The FEM enhances distinctive features within each feature learning network, while the FIM mines the relationships of different features and extracts complementary and salient features across different feature learning networks. Although these modules significantly boost the performance of our method (as shown in Table 4), they also lead to a notable increase in model complexity. Specifically, compared to the baseline method without the MLFMs, the number of parameters in our method is increased by 2 times. Therefore, our model demands higher computation resources and longer training time. To reduce the model complexity while maintaining performance, we will explore reducing the model complexity by decreasing the dimensionality of the input features of the MLFMs. By reducing feature dimensions, we can significantly lower model complexity, particularly for the FIM module (as illustrated in Figure 5). The complexity of weight calculations in our FIM module is proportional to the square of the feature dimension. In future work, we will also investigate implementing the FIM functionality using linearly complex modules.

**Figure 9:**
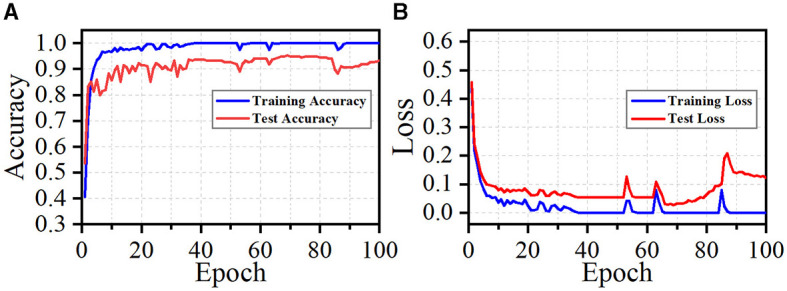
Accuracy **(A)** and loss **(B)** curves during training and test phases.

Although our method achieves superior performance across multiple datasets and exhibits strong generalization ability, it does have limitations. During training, our approach quickly achieves a satisfactory level of performance, as illustrated in Figure 9. We observe that fluctuations in loss and accuracy arise, especially at the later phase of training. It may caused by the great sparsity of the 2D images (as shown in Figure 4). Most regions of these images are background. Noise and background in these images can lead the model to overfit specific morphological features at certain epochs. This suggests that our model is sensitive to particular aspects of the neuron data. To address this, our future work will incorporate data cleaning techniques (Chen et al., 2022; Zhao et al., 2022) and regularization strategies to enhance the robustness of our method. Moreover, higher model complexity coupled with limited data can adversely affect the performance of our model. While dropout, batch normalization, and data augmentation techniques like shifting and flipping are employed in our model, their efficacy is likely suboptimal. Given the tree-like structure of neuronal morphology, tailored data augmentation strategies, as provided in Chen et al. (2022), may yield better results. These include neuron point transformation, branch deformation, and the application of random masks to branches. Monitoring the loss and accuracy curves (as shown in Figure 9) indicates that implementing early stopping could significantly prevent overfitting. Additionally, simplifying our model architecture by pruning layers or reducing feature dimensionality is necessary to enhance the capacity of our model. By introducing these strategies, our model can be a reliable and convenient tool for large-scale neuronal morphology analysis.

## 6 Conclusions

This paper proposes a multi-level feature fusion network for neuronal morphology classification. It thoroughly explored the salient information extracted from hand-crafted morphometrics and 2D images and the shared information between different features through the Multi-Level Fusion Module (MLFM). The MLFM consists of a channel attention-based Feature Enhancement Module (FEM) and a cross-attention-based Feature Interaction Module (FIM). It is embedded in multiple feature extraction blocks to enhance feature learning and facilitate interaction between different features at multiple levels. With the multi-level fusion strategy, the combined neuron descriptors focus on distinguishing spatial features, effectively characterizing neuronal morphology. Our proposed multi-level feature fusion network accurately identifies 10-type neurons with an accuracy of 95.18% and outperforms other methods. Furthermore, the satisfactory performance of the other two datasets demonstrates that our approach can provide a reliable and convenient tool for neuronal morphology analysis.

## Data Availability

The original contributions presented in the study are included in the article/supplementary material, further inquiries can be directed to the corresponding author.

## Footnotes

^1^Data is available in: https://drive.google.com/file/d/1ymYnpMaP6Ocqyllf4JDXKI1slHQtFGDf/view?usp=drive_link.
